# Comparison of short-term outcomes between people with and without a pre-morbid mental health diagnosis following surgery for traumatic hand injury: a prospective longitudinal study of a multicultural cohort

**DOI:** 10.1186/s12891-023-06931-8

**Published:** 2023-10-11

**Authors:** Justine M. Naylor, Pratibha Bhandari, Joseph Descallar, Owen Ou Yang, Mark Rider, Elizabeth C. Mayland, Clarice Tang, Bernadette Brady, David Lim, Yvonne Santalucia, Belinda J. Gabbe, Geraldine Hassett, Elise Baker

**Affiliations:** 1 Orthopaedic Department, Liverpool Hospital, Locked Bag 7103, Liverpool BC, NSW 1871 Australia; 2 Ingham Institute of Applied Medical Research, 1 Campbell St, Liverpool, NSW 2170 Australia; 3 South Western Sydney Hand Centre at Fairfield Hospital, Cnr Polding St. & Prairievale Road, Prairiewood, NSW 2176 Australia; 4 School of Health Sciences, Western Sydney University, Locked Bag 1797, Penrith, NSW 2751 Australia; 5 Department of Pain Medicine, Liverpool Hospital, Locked Bag 7103, Liverpool BC, NSW 1871 Australia; 6 Multicultural Health Service, South West Sydney Local Health District, Locked Bag 7103, Liverpool BC, NSW 1871 Australia; 7 School of Public Health and Preventive Medicine, Monash University, 553 St Kilda Rd, Melbourne, Victoria 3004 Australia; 8 Rheumatology, Liverpool Hospital, Locked Bag 7103, Liverpool BC, NSW 1871 Australia; 9 South Western Sydney Clinical School, Faculty of Medicine, UNSW, Liverpool, BC 1871 NSW Australia; 10 South Western Sydney Local Health District, Liverpool, Locked Bag 7279, Liverpool BC, 1871 Australia

**Keywords:** Traumatic hand injury, QuickDASH, Mental health, Trauma surgery, Culturally and linguistically diverse populations

## Abstract

**Background:**

Following traumatic hand injury, few studies have compared outcomes between people with and without a pre-morbid mental health diagnosis. This study aimed to compare sub-acute outcomes in a multicultural patient cohort with surgically managed traumatic hand injury with and without a pre-morbid mental health diagnosis.

**Methods:**

A prospective, observational cohort study of people with traumatic hand injury presenting pre- surgically to a high-volume hand injury centre in a region of cultural and language diversity was conducted. Participants were assessed face-to-face (baseline) then via telephone (3-months post-surgery) and categorized according to a pre-morbid medically diagnosed mental health diagnosis. Baseline and follow-up assessments included global mental health, and the EuroQol (EQ) ‘Health Today’ analogue scale (0–100) and health domains. Return-to-work status, complications/symptomatic complaints, and hand function (QuickDASH) were also collected at follow-up. Adjusted analyses—accounting for covariates including cultural identity—were conducted to determine whether 3-month outcomes were associated with a pre-morbid mental health diagnosis.

**Results:**

From 405 eligible patients, 386 were enrolled (76% male, mean age 38.9 (standard deviation 15.6)); 57% self-identified as Australian and 22% had a pre-morbid mental health diagnosis. Common injuries regardless of pre-morbid mental health diagnosis were skin (40%), tendon (17%) and bone (17%) injuries. None were complex mutilating injuries. Seventy-eight per cent of the cohort was followed-up. In adjusted analyses, a pre-morbid mental health diagnosis was associated with lower odds for reporting ‘good or better’ global mental health (Odds Ratio (OR) 0.23 (95% Confidence Interval (CI) 0.18, 0.47), *p* < 0.001), ‘no’ anxiety or depression (OR 0.21 (0.11, 0.40), *p* < 0.001) and no pain (OR 0.56 (0.31, 0.98), *p* = 0.04)(EQ domains), and worse EQ ‘Health Today’ (10 points on average (95%CI -14.9, -5.1, *p* < 0.001). QuickDASH scores, rates of complications/symptomatic complaints and return-to-work profiles were similar.

**Conclusions:**

Despite reporting worse mental and health-related quality-of-life outcomes post-surgery, people with a pre-morbid mental health diagnosis regardless of cultural identity experienced similar clinical and return-to-work outcomes. Future research assessing the value of screening for pre-morbid mental health conditions on post-surgical outcomes is required and should include people with more complex hand injuries.

## Background

Mental health symptoms or mood disorders such as depression, anxiety and post-traumatic stress disorders (PTSD) have been observed to be common amongst people with traumatic hand or upper limb conditions [1–8]. Rates vary across the literature, in part due to differing i) tools used to capture mental health profiles (differing patient-reported surveys or use or not of formal diagnoses) [9], ii) diagnostic cut-offs in patient surveys for signifying symptomatic mental health issues [9], iii) injury type and severity, timing of assessment [10], and iv) cohort sample sizes. Though often not accounted for in hand injury populations, rates of mental health conditions may also vary by culture or ethnicity as these diagnoses may be under-recognized or under-reported in ethnic minority groups [11, 12].

A recent review of mental health following upper limb injuries provides estimates ranging 7–71% for depression and/or anxiety and 3–95% for PTSD [10], whilst another review points to the importance of mental health sequelae following upper limb injury [13]. The latter review concluded that the most common factors associated with disability after upper extremity injury or upper extremity pathology are depression, catastrophic thinking, and anxiety, noting also that the magnitude of disability correlates more with the psychosocial constructs than objective measures of impairment [13].

These observations notwithstanding, much of the prior research in this area has focused on the presence of mental health conditions or symptoms following the injury [14], often excluding people with pre-morbid mental health diagnoses [1, 4, 5, 15], or not accounting for pre-morbid mental health diagnoses in the analysis [6, 9, 16]. Whilst the development of mental health conditions or symptoms post-trauma is problematic for functional recovery [4, 5, 9, 13, 17, 18], return to work outcomes [19] and health resource use [17, 20], diagnosed pre-morbid mental health conditions may increase the burden of injury [21], may be associated with more frequent patient complaints [22] and worse clinical outcomes such as digit replant failure [8] and could be associated with concurrent use of drugs of addiction or alcohol which themselves may undermine outcomes [23].

A recent national Australian survey estimated approximately 21% of Australians aged 16–85 reported a mental health or behavioral condition within the previous 12-months [24]. In the United States, amongst a general trauma population categorized by trauma type including those for whom the upper limb injury was self-inflicted (8%), incidences rates of pre-existing psychopathology over 70% were reported [25]. In an African study of work-related upper extremity injuries, the incidence of antecedent mental health conditions was 3% [26]. It is unknown what the rate of pre-morbid mental health diagnoses is amongst traumatic hand injury populations in Australia and whether rates are lower in regions with a high proportion of people from culturally and linguistically diverse (CALD) backgrounds. Further, it is unknown to what extent the pre-morbid mental health diagnosis undermines outcomes.

In light of these knowledge deficits, the aims of this study were to determine the rate of pre-morbid mental health diagnoses in a multicultural cohort of patients with surgically managed traumatic hand injury and compare a range of outcomes in the sub-acute phase between those with and without a pre-morbid mental health diagnosis. We hypothesized that those with a pre-morbid mental health diagnosis would have worse patient-reported health outcomes three months post-surgery after adjusting for covariates including cultural identity.

## Methods

### Design, setting, and ethical approval

A prospective, observational cohort study was undertaken at the South Western Sydney Hand Centre, located within a region which has one of the highest proportions of culturally and linguistically diverse (CALD) communities in Australia [27]. In 2017–18, the rate of mental health disorders in the region (17%) was statistically, significantly lower than the NSW State average (19%) [28], largely attributed to the high proportion of CALD people given the known cultural stigma concerning the presence and reporting of mental health issues [29]. The Centre is multidisciplinary, including medical (surgeons, registrars), allied health, nursing and administrative staff. The Centre predominantly manages patients with fractures and soft tissue injuries of the hand and wrist, wounds to the hand and upper limb, infections and tumors. Referrals to the Centre are received from within the health district from local doctors and hospital emergency departments. Currently, routine mental health assessment either at presentation or at follow-up is not undertaken. In 2021 alone, approximately 5,500 patients were assessed and managed with and without surgery.

The study was approved by the South Western Sydney Human Research Ethics Committee (HREC) (2021/STE02713) as part of the local hand registry (Fairfield Hand Injury & Surgery Registry, FHISt). FHISt is an opt-out registry approved by the HREC where patients presenting to the Centre are informed verbally and via an information sheet (written in English and three community languages (Arabic, Chinese, and Vietnamese)), that routine data collection and follow-up activities will be undertaken as part of quality improvement and research projects.

The STROBE Guidelines were used to report the findings of this study.

### Participant screening and eligibility

All people with traumatic hand injuries, regardless of diagnosis or cause, were screened for eligibility by clinical staff and a research officer (RO) at the time of presentation to the Centre from May to August 2022 inclusive. Those who were ≥ 18 years, could understand written/spoken English, Chinese, Arabic or Vietnamese, and could understand the protocol, were eligible to participate. Those who did not require surgery were ineligible as we specifically aimed to report the mental health profile and outcomes in those undergoing surgery.

Eligible participants who did not opt-out of FHISt were then assessed by the RO or referred to a bilingual Multicultural Health Officer (MHO) for assessment. Eligible participants who did not read English, were contacted by telephone within one week of initial presentation by the MHO trained in the assessment of people from CALD backgrounds, who then completed the translated surveys with the participants in their preferred spoken language.

### Assessment procedure and data collection

At initial presentation (baseline), the RO collected basic sociodemographic information including age, sex, cultural identity, indigenous status, primary language spoken, education, occupation and paid employment status (full, part-time, casual, not working), insurance status (public, private, workers compensation), along with height and weight. Medical history (comorbidities including any medically diagnosed mental health disorders), and use of medication and addiction substances (alcohol, prescription opioids, nicotine and other drugs of addiction), were collected from the patient and verified by medical record review.

Participants were also required to complete an array of patient-reported measures. In all cases, the surveys were administered by the RO or MHO to minimize patient burden given their upper limb impairment and to ensure no survey items were missed. To obtain a pre-injury patient-perceived health profile, each participant completed a series of global questions developed specifically for this study. First, each was asked to rate their global mental health prior to injury on a Likert scale indicating whether their pre-morbid mental health was ‘Excellent’, ‘Very good’, ‘Good’, ‘Neither good nor bad’, ‘Poor’, ‘Very poor’ or ‘Extremely poor’. This global question was then repeated for participants to rate their pre-morbid global health and hand function. Participants also completed a EuroQol-5D-5L (EQ) survey about their current health including the ‘Health Today’ visual analogue scale (VAS) (0–100) and the five associated health domains [30]. Mechanism of injury and hand diagnosis were obtained from the medical record, specifically coded as per routine for hand surgery.

Three months post-surgery, patients were contacted by telephone by the RO or the MHO. The same patient-reported surveys were obtained except the EQ ‘Health Today’ score was necessarily presented verbally. Verbal and visual EQ scales have been shown to be equivalent in a population awaiting arthroplasty surgery [31]. The 11-item QuickDASH Likert scale survey [32] was also used, capturing hand/upper limb symptoms scored 0–100 (lower scores = less symptoms or impairment). Participants were also asked to report any new medically diagnosed mental health disorder in addition to their return- to-work status (full duties, light duties, unable to return), their continued use of opioids at 3-months (if any) and complications or symptomatic complaints (if any). Complications associated with readmission were also verified via review of the medical record.

### Primary, secondary and tertiary outcomes

The primary outcome was patient-perceived global mental health 3-months post-surgery. For reporting and analysis, global mental health was dichotomized into ‘good or better’ and ‘all else’. Secondary outcomes included global health and hand function similarly dichotomized, EQ VAS ‘Health Today’ score, all five EQ domains (dichotomized as ‘No problems’/ ‘None’ vs ‘All else’), and QuickDASH score at 3-months post-surgery. Tertiary outcomes included new medically diagnosed mental health disorders, complications or symptomatic complaints (type and number), continued use of opioids at three months, and return-to-work status.

### Sample size and analyses

A convenience sample of 400 people was planned. Assuming 20% had a pre-morbid mental health diagnosis as per the general community rate, at baseline we anticipated 80 in the pre-morbid mental health diagnosis group and 320 in the group without a pre-morbid diagnosis. A minimum sample of 270 at follow-up (*n* = 45 premorbid mental health diagnosis group; *n* = 225 no premorbid mental health diagnosis), would enable the detection of a 33% relative between-group difference (alpha 0.05) in the primary outcome (60% vs 80%), assuming fewer people in the pre-morbid mental health diagnosis group reported their global mental health to be ‘good or better’. A larger sample at follow-up would enable inclusion of key covariates to determine the adjusted difference. To control for possible confounding, baseline (where measured) values of the outcome of interest were included as dependent variables and adjustments made for covariates (determined a priori) known or postulated to affect patient-reported outcomes including age, sex, insurance status, and cultural identity (dichotomized as ‘Australian’ or ‘Non-Australian’).

Descriptive statistics (mean, standard deviation (sd), proportions (%) and 95% confidence intervals (CI)) were used to profile the cohorts. Baseline between-group comparisons were undertaken using parametric and non-parametric tests as appropriate. Patients lost to follow-up were not analyzed in the 3-month data and no imputation of missing data was undertaken. Comparisons between participants retained and lost to follow-up were undertaken to assess for responder bias.

For the primary outcome, between-group comparisons for the unadjusted and adjusted rates of global mental health (‘good or better’) at 3-months post-surgery were analyzed using Fisher’s exact test and generalized estimating equations (GEE) clustered by participant, respectively. Unadjusted and adjusted analyses for all secondary outcomes were also analyzed using Fisher’s exact test and similar GEE models, respectively. For the GEE models, logit link with binomial distribution was used for dichotomized outcomes, and identity link with Gaussian distribution was used for continuous outcomes. Adjusted Tobit regression was used to analyze the QuickDASH at 3-months necessitated by the high frequency of zero scores (best score), and using the same covariates as for the previously described models. Model residuals were examined to test for normality assumptions where appropriate. Only unadjusted analyses were performed for the tertiary outcomes. *P*-values < 0.05 were interpreted as significantly different with no adjustment for multiple comparisons. Data were entered and stored in RedCap™ by the RO and cleaned and analyzed in SAS Enterprise guide version 8.2 by researchers not associated with data collection.

## Results

### Cohort ascertainment and retention

**Fig. 1:**
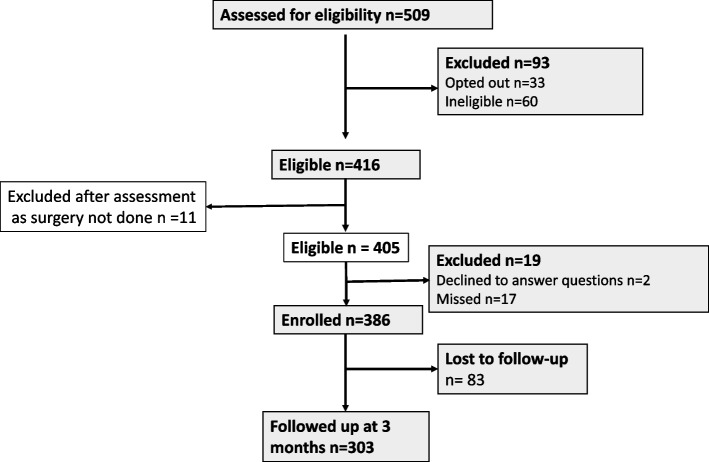
Participant ascertainment and retention

Over the study period, 509 people were screened for eligibility (Fig. 1). Sixty people were ineligible (including 15 due to language) and 33 opted out. After removing people who did not undergo the planned surgery (*n* = 11), 405 remained eligible, but 19 either opted-out later or missed their assessment with the RO. Thus, 386 participants had baseline assessments. At follow-up, the average time between baseline and the 3-month assessment was 90.6 (sd 11.8) days; 83 participants were lost to follow-up leaving 303 (78.5%) with 3-month data. Comparing participants ‘lost’ to those ‘retained’, the groups were similar for most characteristics captured (Table 1), though participants lost to follow-up were significantly younger (mean age 34.5 (sd 12.3) vs 40.2 (sd 16.2) yrs, *p* < 0.001) and more reported to be ‘Indigenous’ (13.3% vs 2.7%, *p* < 0.001).

**Table 1:** Characteristics of retained cohort versus lost to follow-up

	Retained *N* = 303	Lost to follow-up *N* = 83	*p*-value
Age, mean (sd)	40.2 (16.2)	34.5 (12.3)	< 0.001
Male, %	77.9	69.9	0.16
English, primary language, %	76.9	80.7	0.55
Pre-morbid mental health diagnosis, %	22.1	21.7	1.00
Identifies as Australian, %	57.1	57.8	1.00
Identifies as Indigenous,^a^ %	2.6	13.3	< 0.001
Global mental health pre-morbidly, %			
- Good or better^b^	87.8	87.7	1.00
EuroQol ‘Health Today’, mean (sd)	72.4 (18.7)	69.8 (23.0)	0.34

*sd* Standard deviation, *%* Percentage

^a^Aboriginal or Torres Strait Islander

^b^Includes ‘Good’, ‘Very good’, ‘Excellent’

### Cohort characteristics and between-group baseline comparisons

Characteristics of the original cohort are summarized in Tables 2, 3 and 4. The average age was 38.9 (sd 15.6) yrs and the majority (76.2%) were male (Table 2). Most participants were publicly insured (66.3%) and in paid employment (84.5%), and just over half identified as Australian (57.3%). Over one in five (22.3%) reported a pre-morbid mental health diagnosis with the rate varying by cultural identity (29.0% vs 12.7%, Australian vs Non-Australian, *p* < 0.001). Depression was the most common mental health diagnosis in the group reporting a pre-morbid diagnosis (56.5%). Regardless of group, the most common mechanism of injury was soft tissue laceration (43.3%), and the most common surgical diagnosis was skin/soft tissue trauma with no deeper injuries (40%), followed by tendon injury (17%) and bone fracture (17%) (Table 3). No one suffered a complex, mutilating hand injury.

**Table 2:** Sociodemographic characteristics of cohort by pre-morbid mental health diagnosis

	All (*n* = 386)	No pre-morbid mental health diagnosis (*n* = 301)	Pre-morbid mental health diagnosis (*n* = 85)	*p*-value
Age, mean (sd)	38.9 (15.6)	38.7 (16.1)	39.6 (14.1)	0.68
Male, %	76.2	78.4	68.2	0.06
Body mass index, mean (sd)	28.4 (6.3)	28.1 (6.2)	29.6 (6.5)	0.06
Insurance status, %				
- Public	66.3	64.1	74.1	
- Private	10.4	10.6	9.4	0.19
- Workers Compensation	23.3	25.3	16.5	
Paid employment, any (%)	84.5	86.4	77.7	0.06
Type^a^				0.17
- Professional/Office	27.3	25.4	34.9	
- Technician/labourer/	70.6	72.7	62.1	
driver				
- Housekeeping	1.7	1.2	3	
Speak English at home, %	77.7	74.8	88.2	0.008
Self-identify as Australian, %	57.3	52.2	75.3	< 0.001
Addictive drug use, %	9.1	7.7	4.1	0.09
- (Opioid use)	(2.1)	(1.3)	(4.7)	0.08
Education, %				
- No education	2.9	3.4	1.2	0.12
- Up to higher secondary	53.7	51	63.1	
- Tertiary	43.5	45.6	35.7	
Mental health diagnosis, %	22.3			NA
- Depression	12.1		56.5	
- Anxiety	5.1		23.5	
- Anxiety + Depression	4.8		22.4	
- PTSD	2.3		10.6	
- Other	4.9		22.4	
Comorbidity other than mental health diagnosis, %	29.5	26.3	41.2	0.01
Most common comorbidities				
- Hypertension	11.9	12	11.8	1.0
- Asthma	6	4.3	11.8	0.02

*sd* Standard deviation, *%* Percentage

^a^Percentage for ‘Type’ pertains to those in paid employment; *NA* Not applicable, *PTSD* Post-traumatic stress disorder

**Table 3:** Mechanism of injury and injury diagnoses

	All	No pre-morbid mental health diagnosis *N* = 301	Pre-morbid mental health diagnosis *N* = 85	*p*-value
**Mechanism of injury, %**				
Laceration	43.3	41.9	48.2	0.32
Workplace	19.7	20.9	15.3	0.28
Power tool	12.7	14.0	8.2	0.20
Sport	6.0	6.3	4.7	0.80
Bite	5.7	4.7	9.4	0.11
Motor bike accident	2.6	2.7	2.4	1.0
Self-harm	0.8	0.7	1.2	0.52
Motor vehicle crash	0.5	0.3	1.2	0.39
Mower	0.5	0.7	0	1.0
High pressure	0.3	0.3	0	1.00
Other	11.4	11.6	10.6	1.0
**Diagnosis, %**				
Soft tissue laceration	40	40	39	0.80
Tendon rupture (any)	17	18	14	0.42
Fracture (any)	17	17	14	0.62
Nail bed	17	17	16	1.0
Nerve dissection	5	5	5	1.0
Artery dissection	5	6	4	0.59
Amputation (any)	3	3	2	1.0
Dislocation (any)	1	1	0	0.58
Injury requiring a graft	1	1	2	0.30
Other^a^	14	14	16	0.49

*%* Percentage

^a^Includes various unique injuries such as damage to finger pulp, infection, nerve compression, granuloma, bite. (Note: Diagnoses are not mutually exclusive as multiple injuries possible)

**Table 4:** Patient-reported health pre-injury and on presentation

	No pre-morbid mental health diagnosis *N* = 301	Pre-morbid mental health diagnosis *N* = 85	*p*-value
Global mental health, %			
- Good or better^a^	94.7	63.5	< 0.001
- All else^b^	5.3	36.5	
Global hand function, %			
- Good or better	98.7	94.1	0.03
- All else	1.3	5.9	
Global health, %			
- Good or better	97.0	87.1	0.001
- All else	3.0	12.9	
EuroQol VAS Health Today, mean (sd)	73.4 (19.3)	66.1 (20.2)	0.003
EuroQol Mobility problems, %			
- No problems	95.7	89.4	0.04
- Any problems^c^	4.3	10.6	
EuroQol Personal Care problems, %			
- No problems	32.3	30.6	0.79
- Any problems	67.7	69.4	
EuroQol Usual Activities problems, %			
- No problems	20.3	10.6	0.05
- Any problems	79.7	89.4	
EuroQol Pain/Discomfort symptoms, %			
- No pain	15.6	11.8	0.49
- Any symptoms	84.4	88.2	
EuroQol Anxiety/Depression symptoms, %			
- No symptoms	69.4	44.7	< 0.001
- Any symptoms^c^	30.6	55.3	

*sd* Standard deviation, *%* Percentage

^a^Includes ‘Excellent’, ‘Very good’, ‘Good’

^b^Includes ‘Neither’, ‘Poor’, ‘Very poor’ or ‘Extremely poor’

^c^Includes ‘Slight’, ‘Moderate’, ‘Severe’, ‘Extreme’

In addition, the group with a pre-morbid mental health diagnosis had a significantly higher percentages of participants identifying as Australian (75.3% vs 52.2%), and having a comorbidity (other than a mental health diagnosis) (41.2% vs 26.3%) (Table 2). Hypertension and asthma were the two most common comorbidities in either group with those in the pre-morbid mental health group having a higher incidence of asthma (11.8% vs 4.3%, *p* = 0.02). Baseline (pre-injury or day of presentation) patient-reported outcome measures (PROMs) are summarized in Table 4. The group with a pre-morbid mental health diagnosis scored significantly worse in PROMs for all global questions, the EQ VAS ‘Health Today’ score and two EQ health domains (Mobility; Anxiety or Depression).

### Outcomes at 3-months

#### New medically diagnosed mental health disorder

One participant in each group reported a newly diagnosed mental health disorder at 3-months (0.4% vs 1.5%, *p* = 0.04, ‘No pre-morbid mental health diagnosis’ group vs ‘Pre-morbid mental health diagnosis’ group), with one participant in the ‘No’ group reporting a diagnosis of depression/anxiety and another in the ‘Pre-morbid mental health diagnosis’ group reporting post-traumatic stress disorder.

#### Primary outcome—Global mental health

In both unadjusted and adjusted comparisons, significantly fewer participants in the ‘Pre-morbid mental health diagnosis’ group reported ‘good or better’ global mental health at 3-months (Table 5) than those in the ‘No pre-morbid mental health diagnosis’ group. Specifically, those with a pre-morbid mental health diagnosis had reduced odds (OR 0.23 (0.18, 0.47), *p* < 0.001) of reporting good or better mental health.

**Table 5:** Unadjusted and adjusted patient-reported health and upper limb outcomes at 3-months

	No pre-morbid mental health diagnosis *N* = 236	Pre-morbid mental health diagnosis *N* = 67	*P*-value (unadjusted)	Odds Ratio or beta coefficient^b^ (95% CI) (adjusted)	*p*-value (adjusted)
Global mental health, %					
- Good or better^a^	90.3	68.6	< 0.001	0.23 (0.18, 0.47)	< 0.001
Global hand function, %					
- Good or better	86.0	86.6	1.0	0.91 (0.39, 2.15)	0.84
Global health, %					
- Good or better	94.5	89.6	0.17	0.45 (0.16, 1.28)	0.13
EQ VAS Health Today, mean (sd)	83.7 (17.7)	73.6 (18.2)	< 0.001	-10.02 (-14.9, -5.1)	< 0.001
EQ Mobility					
- No problems	96.2	93.9	0.49	0.55 (0.17, 1.81)	0.38
EQ Personal Care					
- No problems	91.1	78.8	0.009	0.36 (0.17, 0.76)	0.008
EQ Usual Activities					
- No problems	82.6	74.2	0.16	0.58 (0.30,1.14)	0.12
EQ Pain					
- None	72.5	59.7	0.05	0.55 (0.31, 0.98)	0.043
EQ anxiety/depression					
- None	89.0	62.7	< 0.001	0.21 (0.11, 0.40)	< 0.001
Quickdash^c^, mean (sd)	11.2 (17.9)	15.2 (18.8)	0.12	6.65 (0.-0.84, 14.2)	0.08

*%* Percentage, *EQ* Euroqol-5D-5L, *sd* Standard deviation, *CI* Confidence interval

^a^Includes ‘Good’, ‘Very good’, ‘Excellent’

^b^Odds ratio (dichotomous) or beta coefficient (continuous) is for pre-morbid mental health diagnosis vs no pre-morbid mental health diagnosis. Generalized estimating equations (GEE) were used to model the PROM scores from baseline to 3 months, using patient as a cluster variable, and accounting for pre-morbid mental health diagnosis (yes/no), age, sex, insurance status, cultural identity, and related baseline PROM except for QuickDASH (not captured at baseline). Tobit regression was used to model Quickdash

^c^Quickdash, Quick Disabilities of the arm, shoulder, and hand questionnaire

#### Secondary outcomes

In both unadjusted and adjusted comparisons, EQ ‘Health Today’ was significantly worse in the ‘Pre-morbid mental health diagnosis’ group by 10 points (unadjusted -10.1, *p* < 0001; adjusted -10.02 (95% CI-14.9, -5.1), *p* < 0.001). In both unadjusted and adjusted comparisons, participants in the ‘Pre-morbid mental health diagnosis’ group less frequently reported or were less likely to report ‘No problems’ in the EQ Personal Care, and ‘None’ in the Pain and Anxiety or Depression domains (Table 5); global hand and global health outcomes were not different. In adjusted analysis, the QuickDASH score was higher (worse) in the ‘Pre-morbid mental health diagnosis’ group (Table 5) on average by ~ 7 points, but the difference did not reach statistical significance (6.65 (95% CI -0.84, 14.2), *p* = 0.08).

#### Tertiary outcome

Complications/symptomatic complaints (type and number) were similar between the two groups (Table 6). Stiffness or range of motion concerns were the most common (10.5% vs 11.8%, ‘Pre-morbid mental health diagnosis’ vs ‘No Pre-morbid mental health diagnosis’ group). Return-to-work rates and rates of continued opioid use at three months were also similar (Table 6).

**Table 6:** Complications/symptomatic complaints and return-to-work outcomes at 3-months

	No pre-morbid mental health diagnosis *N* = 236	Pre-morbid mental health diagnosis *N* = 67	*p*-value
Complications and symptomatic complaints			
Any, %	16.9	19.4	0.72
Stiffness/Range of motion issues (%)	11.8	10.5	1
Hand, upper limb strength, %	4.2	4.5	1
Hand pain, %	2.1	4.5	0.38
Other, %	0.7	2.4	0.21
Return-to-work outcomes^a^			
Full duties, %	79.0	77.6	0.85
Light duties, %	10.3	12.2	0.8
Ongoing opioid use^b^			
Yes, %	5.2	13.2	0.14

*%* Percentage

^a^Denominator includes only those who were in paid employment at presentation [*n* = 195 in No pre-morbid mental health diagnosis group; *n* = 49 in pre-morbid mental health group]

^b^Sample for whom this question was asked was reduced (*n* = 116, No pre-morbid mental health diagnosis; *n* = 38, Pre-morbid mental health diagnosis)

## Discussion

In a patient population with a diverse range of surgically managed hand trauma, where one in five speak a language other than English at home, and 43% identify as non-Australian, a pre-morbid or antecedent diagnosed mental health disorder was at least as common as that seen in the general population [24] and possibly more common than in the immediate surrounding community [28]. Consistent with our hypothesis, the presence of a pre-morbid health diagnosis appears to systemically undermine recovery across some patient-reported health domains (general health and mental health) and this does not appear to be explained by differences in injury type, complications or symptomatic complaints, return-to- work outcomes, baseline PROM scores (where included in adjusted analyses), age, sex, insurance status and cultural identity. Interestingly, other hand-specific clinical outcomes and RTW outcomes were not associated with a pre-morbid mental health diagnosis.

Whilst our rate of antecedent mental health diagnoses appears high compared to that reported for the region, in contrast to other studies [4, 6, 33], our rate of diagnosed mental health disorders is comparatively low even accounting for the few patients who reported a new mental-health diagnosis across time. Though not quantified in this study, a comparatively low level of injury severity may partly explain this observation given we had mainly non-specific soft tissues injuries secondary to laceration (as opposed to mutilation and amputation-type injuries secondary to crush injury or major trauma) and that the QuickDASH outcomes at three months point to low-level impairment in general. Further, most studies reporting mental health across time rely on patient reported surveys [9, 14], not medically diagnosed conditions, thus highlighting the problem of differing methods of ascertainment clouding the determination of the true incidence of diagnosed mental health conditions. Alternatively, or perhaps additionally, the ethnic diversity in our study may be contributing to our comparatively low rate of mental health diagnoses. We note the rate of pre-morbid mental health conditions was much lower amongst participants (almost half the cohort) who did not identify as Australian. It is known that mental health stigma is higher among racial or ethnic minorities, thus, some cultural groups may under-report or under-recognize mental health complaints [11, 12, 29, 34]. Alternatively or additionally, there may be a healthy immigrant effect but we did not collect country of birth, thus, we are unable to compare health profiles of those born locally versus overseas.

Many previous investigators have called for mental health screening and psychosocial services to be part of standard care for this patient population [9, 13, 16, 20, 21, 23, 35, 36], but recent research suggests that such screening or services are not common amongst any hand trauma services [37]. Our study brings new knowledge to this field providing strong support for new models of care which recognize the associated mental health challenges. That recovery in patient-reported mental health and quality of life remains inferior for those with a pre-morbid mental health diagnosis even after adjustment for key factors, suggests there may be a role for care providers or models in reducing these gaps. Further, our observation that the rate of pre-morbid mental health diagnosis amongst those who did not identify as Australian was less than half that of those who did, suggests that for services with a high proportion of CALD patients, culturally responsive pathways that enable such patients to communicate freely about any mental health concerns should be included so potentially “undiagnosed” mental health conditions are not overlooked.

### Strengths and limitations

Our study has several strengths. It appears to be one of the few and largest prospective studies available exploring the relationship between medically diagnosed pre-morbid mental health conditions and outcomes following surgery for traumatic hand injury, whilst accounting for cultural identity and other important factors. Though our follow-up time was limited, 3-months is considered sufficient for detecting PTSD, anxiety, depression and chronic pain in a hand cohort [9]. Our findings are generalizable to heterogeneous populations of varying ethnicity and primary languages given our broad inclusion criteria and use of interpreters and translated documents.

We also recognize some potential limitations. Our study could not differentiate between poorly or well managed mental health diagnoses, thus, our binary categorization could mask different recovery trajectories based on how well pre-morbid mental health conditions were managed. Our results are also likely only generalizable to cohorts manifesting mild-to-moderate traumatic hand injury requiring surgery. That our ‘lost’ cohort were younger and more likely to identify as ‘indigenous’ means our findings may not generalize to these sub-groups. We did not adjust for multiple comparisons though the consistency in the directional changes (poorer scores in the pre-morbid mental health group across all but one (global hand function) patient-reported outcomes), would suggest the differences are not likely to be random. Finally, the global outcome questions used have not undergone clinimetric testing, thus, their validity, reliability and sensitivity are assumed. We note, however, that the global mental health question appears to exhibit face validity given the pre-morbid mental health group (identified independently using their medical history) reported worse global mental health at baseline compared to the group without a mental health history as would be expected.

## Conclusions

In a cohort of people who experienced traumatic hand injury (excluding complex mutilating injuries) regardless of cultural identity, those with a pre-morbid mental health diagnosis reported worse mental and health-related quality of life outcomes post-surgery compared to those without such diagnoses. Surprisingly, hand-specific clinical outcomes and return-to-work profiles were similar. Future research assessing the value of screening for pre-morbid mental health conditions on post-surgical outcomes is required and should include people with more complex hand injuries.

## Data Availability

The datasets used during the current study are available from the corresponding author on reasonable request. The datasets have not been made publicly available due to the risk of reidentification of individuals owing to a combination of a small sample size (< 500), the hospital is identifiable, the surgery date range is known, and some rare diagnoses are present.

## Abbreviations

%: Per cent or percentage
CI: Confidence interval
EQ: EuroQol-5D-5L
FHISt: Fairfield Hand Injury & Surgery Registry
GEE: Generalized estimating equation
MHO: Multicultural Health Officer
*p*-value: Probability value
Quickdash: Quick Disabilities of the arm, shoulder, and hand (questionnaire)
RO: Research officer
sd: Standard deviation
VAS: Visual analogue scale
